# Genotoxic, Cytotoxic, Antigenotoxic, and Anticytotoxic Effects of Sulfonamide Chalcone Using the Ames Test and the Mouse Bone Marrow Micronucleus Test

**DOI:** 10.1371/journal.pone.0137063

**Published:** 2015-09-03

**Authors:** Carolina Ribeiro e Silva, Flávio Fernandes Veloso Borges, Aline Bernardes, Caridad Noda Perez, Daniela de Melo e Silva, Lee Chen-Chen

**Affiliations:** 1 Instituto de Ciências Biológicas, Universidade Federal de Goiás, Goiânia, Goiás, Brazil; 2 Instituto de Química, Universidade Federal de Goiás, Goiânia, Goiás, Brazil; Panjab University, INDIA

## Abstract

Chalcones present several biological activities and sulfonamide chalcone derivatives have shown important biological applications, including antitumor activity. In this study, genotoxic, cytotoxic, antigenotoxic, and anticytotoxic activities of the sulfonamide chalcone N-{4-[3-(4-nitrophenyl)prop-2-enoyl]phenyl} benzenesulfonamide (CPN) were assessed using the *Salmonella typhimurium* reverse mutation test (Ames test) and the mouse bone marrow micronucleus test. The results showed that CPN caused a small increase in the number of histidine revertant colonies in *S*. *typhimurium* strains TA98 and TA100, but not statistically significant (*p* > 0.05). The antimutagenicity test showed that CPN significantly decreased the number of His^+^ revertants in strain TA98 at all doses tested (*p* < 0.05), whereas in strain TA100 this occurred only at doses higher than 50 μg/plate (*p* < 0.05). The results of the micronucleus test indicated that CPN significantly increased the frequency of micronucleated polychromatic erythrocytes (MNPCE) at 24 h and 48 h, revealing a genotoxic effect of this compound. Also, a significant decrease in polychromatic/normochromatic erythrocyte ratio (PCE/NCE) was observed at the higher doses of CPN at 24 h and 48 h (*p* < 0.05), indicating its cytotoxic action. CPN co-administered with mitomycin C (MMC) significantly decreased the frequency of MNPCE at almost all doses tested at 24 h (*p* < 0.05), showing its antigenotoxic activity, and also presented a small decrease in MNPCE at 48 h (*p* > 0.05). Additionally, CPN co-administered with MMC significantly increased PCE/NCE ratio at all doses tested, demonstrating its anticytotoxic effect. In summary, CPN presented genotoxic, cytotoxic, antigenotoxic, and anticytotoxic properties.

## Introduction

Chalcones are natural compounds of widespread occurrence in plants and considered precursors of flavonoids. They can be synthesized by Claisen-Schmidt condensation between benzaldehydes and acetophenones using either basic or acidic catalysis [1,2]. These compounds have been reported to exhibit a wide range of pharmaceutical effects, including antioncogenetic, anti-inflammatory, anti-ulcerative, antimalarial, antiviral, antifungal, and antibacterial activities [3]. Also, some of their derivatives have proven to be antimutagenic [4].

It is well known that several substituents, in different positions on the core structure of chalcones, can determine different biological activities as well as specificity of action [5]. Recently, sulfonamide chalcone derivatives have received significant attention due to their fungicidal, antimalarial, antileishmanial, and antitumor properties, as well as inhibitory activity against α-glycosidase [6–8]. Considering the importance of chalcones and sulfonamides for further therapeutic development, it is relevant to evaluate the genotoxic and antigenotoxic activities of these compounds.

The search for genotoxic and antigenotoxic compounds and the evaluation of their mechanisms of action deserve special attention due to their potential role in the protection of human health [9]. *In vitro* and *in vivo* genotoxicity tests are used to assess the capability of new chemicals to cause damage to DNA, since they allow the examination of gene mutations and chromosomal alterations [10]. Also, these tests are employed in studies that evaluate the antigenotoxic potential of chemical compounds.

Short-term genotoxicity assays have been used for screening mutagens and potential carcinogens in human environments, as well as for the identification of antimutagens and anticarcinogens. One of these assays, the Ames mutagenicity test, uses several *Salmonella typhimurium* tester strains to measure two classes of gene mutation, namely base pair substitutions and small frameshifts [11]. This test serves as a model for predicting and understanding the toxicological properties of tested substances [12]. Another well-known genotoxic assay, the mouse bone marrow micronucleus test, has proven suitable for the evaluation of genotoxic and antigenotoxic actions of compounds [13,14]. Additionally, the frequency of micronucleated polychromatic erythrocytes (MNPCE) is a reliable measure of both chromosome loss and breakage [15].

Given that chalcones and sulfonamides present several biological activities and that the knowledge of their genotoxic and antigenotoxic effects is limited, the aim of this study was to evaluate the genotoxic, cytotoxic, and protective effects of the sulfonamide chalcone N-{4-[3-(4-nitrophenyl)prop-2-enoyl]phenyl}-benzenesulfonamide (CPN) using the *S*. *typhimurium* reverse mutation assay (Ames test) and the mouse bone marrow micronucleus test.

## Materials and Methods

### Synthesis of compounds

**Fig 1:**
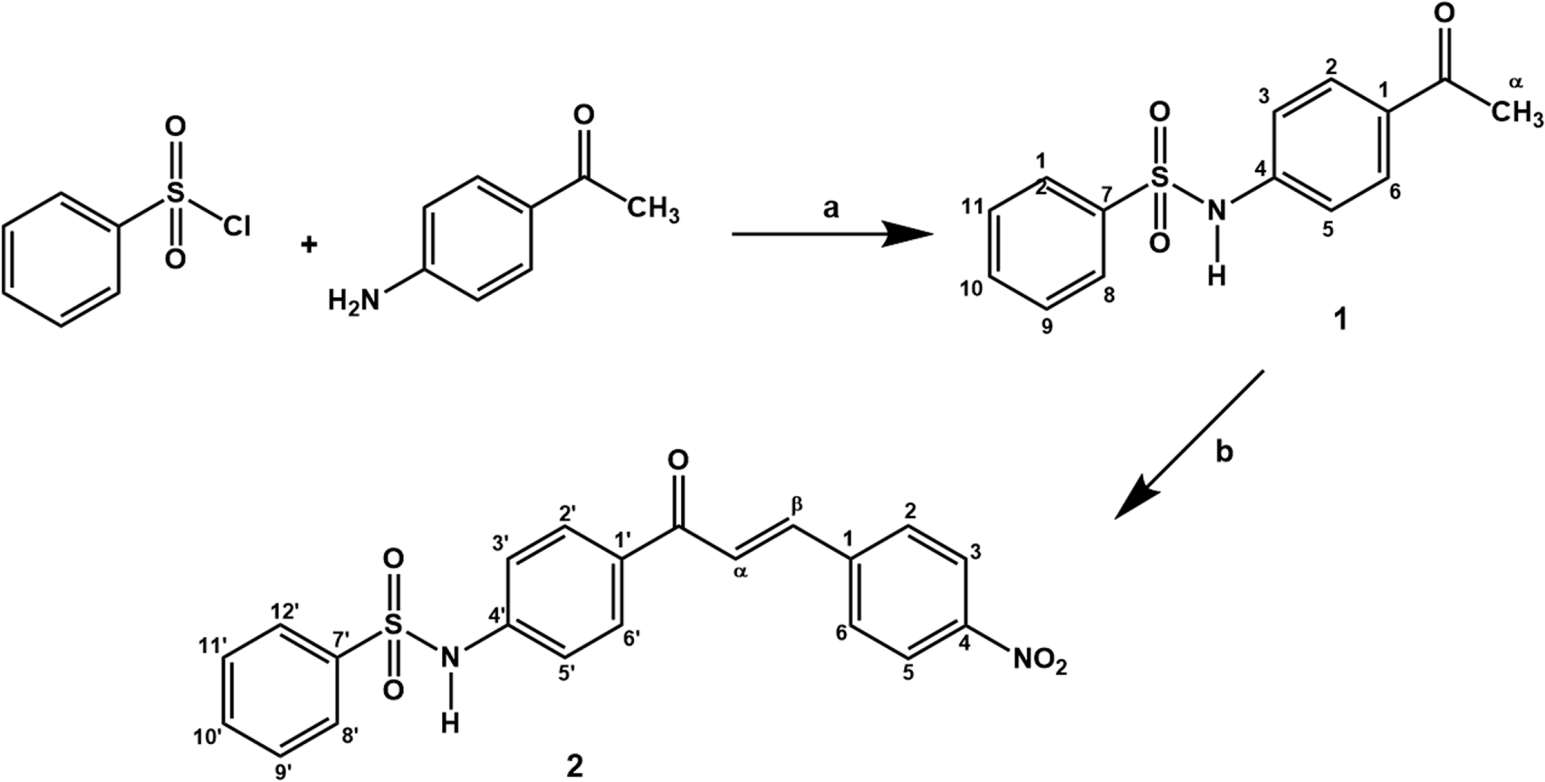
Synthetic route of sulfonamide chalcone (2) and its precursor, N-(4-acetylphenyl)benzenesulfonamide (1). Reagents and conditions: (a) CH_2_Cl_2_, 6 h (reflux); (b) *p*-nitrobenzaldehyde, EtOH, NaOH (50% w/w, ketone) 24 h (room temperature).

All commercially available reagents were purchased from Sigma-Aldrich (St. Louis, MO, USA). ^1^H NMR spectra were recorded on a Bruker Avance III (500 MHz) spectrometer (Bruker Optik GmbH, Ettlingen, Germany). Infrared (IR) spectra were performed on a Bomem M102 spectrometer (Bomem Inc., Vanier, Quebec, Canada). Mass spectra were recorded on a Shimadzu GCMS-QP2000 (Shimadzu do Brasil Comércio Ltda., São Paulo, SP, Brazil). The melting points were measured using a melting point apparatus (Karl Kolb GmbH & Co., Dreieich, Germany). The purity of compounds was determined by HPLC (> 99%) using Waters Alliance—2695 apparatus, XTerra C18 (5 μm, 4.6 mm x 150 mm) column, Waters 2998 Photodiode Array (PAD) detector at 273 nm and 322 nm for quantification of analyte 1 (benzenesulfonamide derivative) and 2 (sulfonamide chalcone), respectively. The eluent flow rate was 1.5 mL/min and the injector was programmed to inject a volume of 10 μL. The mobile phase was CH_3_CN:aqueous buffer containing chloroacetic acid 0.1% (55:45). The synthesis of the designed compounds is outlined in Fig 1.

#### Synthesis of benzenesulfonilamide derivative (1)

The mixture of phenylsulfonyl chloride (1.0 mmol) and *p*-aminoacetophenone (1.0 mmol) was refluxed in 5 mL of dichloromethane for 6 h while stirring. The reaction mixture was cooled using an ice bath and the solvent was removed under reduced pressure. The precipitate was washed with hexane, dried, and purified by recrystallization from acetone/water (2:1). The yield of the synthesis, physicochemical properties, and purity of compound 1 are listed in Table 1.

**Table 1:** Yield, physical state, melting point, purity, and spectroscopic data (GS-MS, IR, ^1^H-NMR) of N-(4-acetylphenyl)benzenesulfonamide (1) and N-{4-[(*E*)-3-(4-nitrophenyl)prop-2-enoyl]phenyl}-benzenesulfonamide (2).

Parameter	Compound 1	Compound 2
Yield (%)	54	55
Physical state	Yellowish white crystal	Orange crystal
Melting point (°C)	113–115	178–181
Purity in HPLC (RT)^1^	100% (1.953 min)^2^	99.68% (5.022 min)^2^
GC-MS data	275 [M^+^] (calculated for C_14_H_12_NO_3_S, 275.062)	408 [M^+^] (calculated for C_21_H_16_N_2_O_5_S, 408.078)
IR (KBr) (cm^-1^)	3219 (ν N-H), 1674 (ν C = O), 1593 (ν N-H), 1513 (ν C = O), 1337 (ν S = O), 1277 (ν C-N), 1155 (ν S = O)	3211 (ν N-H), 1658 (ν C = O), 1607 (ν Ar-CO-C = C-Ar), 1516 (ν N-H), 1342 (ν S = O), 1281, 1223 (ν C-N), 1155 (ν S = O)
1H-NMR (500 MHz-DMSO-d^6^) δ	2.46 (*s*, 3H, H*α*), 7.21 (*ddd*, 2H, *J* = 8.7, *J* = 9.2, H3, H5), 7.57 (*t*, 2H, *J* = 7.9, H9, H11), 7.63 (*t*, 1H, *J* = 7.9, H10), 7.81–7.84 (*m*, 4H, H2, H6, H8, H12)	7.22 (*ddd*, 2H, *J* = 8.9, *J* = 9.3, H3’, H5’), 7.42 (*dt*, 2H, *J* = 7.4, *J* = 1.2, H9’, H11’), 7.55–7.60 (*d*, *dt*, 2H, *J* = 15.8, *J* = 7.4, *J* = 1.2, H*α*, H10’), 7.77 (*ddd*, 2H, *J* = 8.8, *J* = 9.2, H2, H6), 7.80 (*d*, 1H, *J* = 15.8, H*β*), 7.86 (*dt*, 2H, *J* = 8.1, *J* = 2.1, *J* = 1.2, H8’, H12’), 7.96 (*ddd*, 2H, *J* = 8.9, *J* = 9.3, H2’, H6’), 8.28 (*ddd*, 2H, *J* = 8.8, *J* = 9.2, H3, H5)

^1^ Retention time.

^2^ Mobile phase used: CH_3_CN:aqueous buffer containing chloroacetic acid 0.1% (55:45).

#### Synthesis of sulfonamide chalcone (2)

Sulfonamide chalcone was synthesized by Claisen-Schmidt condensation. Equimolar portions of N-(4-acetylphenyl)benzenesulfonamide (1.0 mmol) and *p*-nitrobenzaldehyde (1.0 mmol) were added to 30 mL of 50% NaOH ethanol solution (w/w). The reaction mixture was stirred at room temperature for 24 h, poured into cold water (20 mL), and neutralized with 10% hydrochloric acid (w/v), forming an orange-yellow solid compound, which was extracted three times with dichloromethane. The organic phases were combined, washed with water, separated, and evaporated. The resulting solid compound was purified by recrystallization from acetonitrile/ethyl acetate (1:3).

Table 1 shows the yield of the synthesis, physicochemical properties, and purity of compound 2. A coupling constant of *ca*.15.8 Hz for the vinyl H-atoms in the ^1^H-NMR spectra confirmed the *(E)-*configuration of this compound.

### Ames test

#### Strains

*Salmonella typhimurium* tester strains TA98 and TA100 were kindly supplied by Divisão de Toxicologia, Genotoxicidade e Microbiologia Ambiental of Companhia de Tecnologia de Saneamento Ambiental do Estado de São Paulo (CETESB, São Paulo, SP, Brazil).

#### Experimental procedure

The *S*. *typhimurium* histidine point mutation assay proposed by Maron and Ames [16] was followed. The doses of CPN used in this assay were 1, 10, 20, 50, 100, 200, 500, 1000, 2000, and 5000 μg/plate and each dose was diluted in 20 μL of dimethylsulfoxide (DMSO, lot no. 0900221, Vetec, Duque de Caxias, RJ, Brazil). A 0.1-mL aliquot of bacterial suspension (1–2 × 10^9^ cells/mL) of each strain (TA98 and TA100) was incubated with the different doses of CPN at 37°C for 25 min. A 2.0-mL aliquot of top agar (0.6% Difco agar, 0.5% NaCl, 50 μM L-histidine, and 50 μΜ biotin, at 45°C) was added to the test tubes and poured onto Petri dishes containing minimal agar medium (1.5% agar, 2% glucose, and Vogel-Bonner medium E). Each assay was performed three times in triplicate and included a negative control (20 μL DMSO), and a positive control, 0.5 μg 4-nitroquinoline 1-oxide (4-NQO)/plate (lot no. SLBD6960V, Sigma-Aldrich, São Paulo, SP, Brazil) for TA98, and 1.5 μg sodium azide/plate (lot no. 26628–22–8, Merck, Cotia, SP) for TA100. For the antimutagenicity evaluation, the same doses of CPN employed in the mutagenic evaluation were co-treated with their respective positive controls. His^+^ revertant colonies were counted after incubation for 48 h at 37°C.

#### Statistical analysis

Statistical analysis was carried out using Excel® and/or SPSS 20 and the results were expressed as mean ± standard deviation (SD). ANOVA and the Tukey’s test were used to evaluate the significant difference between treatments and control groups. P values lower than 0.05 (*p* < 0.05) were considered indicative of significance. The magnitude of the mutagenicity induction was measured by the mutagenic index (MI), calculated as the ratio between the number of colonies in the test treatment and the number of colonies in the negative control treatment. In order to evaluate the antimutagenicity, the inhibition percentage (IP) of mutagenicity induced by the mutagen was calculated using the following formula [17]:

$$IP(\%)=\left[1-\left(\frac{TP-SR}{PCP-SR}\right)\right]\times 100$$

where:

TP: number of His^+^ revertants on test plates (plates incubated with mutagen and compound)

PCP: number of His^+^ revertants on positive control plates (plates incubated with the mutagen alone)

SR: spontaneous His^+^ revertants (tester strains incubated in the absence of compound and mutagen)

### Mouse bone marrow micronucleus test

#### Animals

The present study was approved by the Human and Animal Research Ethics Committee of the Universidade Federal de Goiás (CoEP/UFG no. 017 /2011). Healthy, young, male, adult (8–12 weeks) outbred mice (*Mus musculus*, Swiss Webster), weighing 25–30 g, obtained from the Central Animal House of the Universidade Federal de Goiás, were randomly allocated to treatment groups. All animals were brought to the laboratory five days before the experiments and housed in plastic cages (40 cm × 30 cm × 16 cm), lined with wood shavings, kept at 24 ± 2°C and 55 ± 10% humidity, with a light-dark natural cycle of 12 h. Food (commercial rodent diet Labina, Ecibra Ltda., Santo Amaro, SP, Brazil) and water were given *ad libitum*.

#### Experimental procedure

The experiments were carried out according to von Ledebur and Schmid [18]. For the genotoxic assessment, four doses of CPN (25, 50, 100, and 150 mg/kg body weight—bw) were intraperitoneally (i.p.) administered to groups of five animals for each treatment. The same doses were co-administered with mitomycin C (MMC, 4 mg/kg i.p.– 80% LD50, lot no. 1K00322, Bristol, Mayers Squibb, São Paulo, SP, Brazil) to groups of five animals per treatment to perform the antigenotoxicity evaluation. A positive control group (MMC, 4 mg/kg i.p.– 80% LD50) and a negative control group (DMSO, 0.1 mL/10 g bw i.p.) were included.

The animals were euthanized by cervical dislocation 24 h or 48 h after the administration of CPN, and their bone marrow cells were flushed from both femurs in fetal calf serum (FCS, lot no. 61005001, Laborclin, Pinhais, PR, Brazil). After centrifugation (300× g, 5 min), the bone marrow cells were smeared on glass slides, coded for blind analysis, air-dried, and fixed with absolute methanol (CH_4_O, lot no. 1207433COD: 000102.06, Vetec, Duque de Caxias, RJ, Brazil) for 5 min at room temperature. The smears were stained with Giemsa (lot no. 21101108C, Newprov, Pinhais, PR, Brazil), dibasic sodium phosphate (Na_2_HPO_4_12H_2_O, lot no. 982162, Vetec, Duque de Caxias, RJ, Brazil), and monobasic sodium phosphate (NaH_2_PO_4_H_2_O, lot no. 983831, Vetec, Duque de Caxias, RJ, Brazil) to detect micronucleated polychromatic erythrocytes (MNPCE).

For each animal, four slides were prepared and 2000 polychromatic erythrocytes (PCE) were counted to determine the frequency of MNPCE using light microscopy (Olympus BH-2 10 × 100, Tokyo, Japan). Genotoxicity and antigenotoxicity were assessed by the frequency of MNPCE, whereas cytotoxicity and anticytotoxicity were evaluated by the PCE and normochromatic erythrocytes (NCE) ratio (PCE/NCE).

#### Statistical analysis

To analyze the genotoxic and antigenotoxic activities of CPN using the mouse bone marrow micronucleus test, the frequency of MNPCE in the treated groups was compared to the results obtained for the negative control group applying one-way ANOVA and Tukey’s post hoc test. To assess its cytotoxicity and anticytotoxicity, the PCE/NCE ratio of CPN was compared to the negative control using the chi-square (χ^**2**^) test. P values lower than 0.05 (*p* < 0.05) were considered indicative of significance.

## Results

### Ames test

The results of the mutagenic and antimutagenic evaluation, in three independent experiments carried out in triplicate, are shown in Table 2. The data obtained for the positive and negative control groups indicated that the strains were in agreement with the guidance established by Maron and Ames [16] and Mortelmans and Zeiger [11].

**Table 2:** Means ± standard deviation (SD) of histidine revertant colonies, mutagenic index (MI), and inhibition percentage (IP) of mutagenicity for two tester strains of *Salmonella typhimurium*, TA98 and TA100, after treatment with different doses of sulfonamide chalcone N-{4-[3-(4-nitrophenyl)prop-2-enoyl]phenyl}-benzenesulfonamide (CPN).

Treatment	Mutagenicity^3^				Antimutagenicity^3^			
	TA98		TA100		TA98		TA100	
	Mean±SD	MI	Mean±SD	MI	Mean±SD	IP (%)	Mean±SD	IP (%)
Negative control^1^	24.8±6.0^a^ ^,^ ^b^	1.00	202.0±61.0^a^	1.00	25.9±7.4^a^	–	174.0±38.0^a^	–
Positive control^2^	377.4±27.0^c^	15.22	1822.3±353.1^b^	9.02	383.4±63.5^b^	–	2086.8±196.5^b^	–
CPN 1 μg/plate	29.0±7.0^a^	1.17	201.7±57.6^a^	0.99	213.7±31.8^c^	47	1890.5±241.6^b^	10
CPN 10 μg/plate	30.8±7.7^a^	1.24	214.0±65.5^a^	1.06	201.9±49.3^c^	51	1830.0±123.0^b^	13
CPN 20 μg /plate	38.4±8.8^a^	1.55	238.0±41.0^a^	1.18	203.9±47.9^c^	50	1834.0±29.9^b^	13
CPN 50 μg/plate	28.3±6.1^a^	1.14	235.3±54.6^a^	1.16	207.8±27.7^c^	49	1704.3±157.8^b^ ^,^ ^d^	20
CPN 100 μg/plate	30.7±7.2^a^	1.24	254.7±49.2^a^	1.26	208.7±44.1^c^	49	1610.0±136.3^c^ ^,^ ^d^	25
CPN 200 μg/plate	32.5±6.2^a^	1.31	235.7±16.2^a^	1.17	203.2±22.9^c^	50	1556.3±137.1^c^ ^,^ ^d^	28
CPN 500 μg/plate	35.2±5.5^a^	1.42	260.9±35.2^a^	1.29	198.2±20.8^c^	52	1525.0±104.6^c^ ^,^ ^d^	29
CPN 1000 μg/plate	39.4±3.9^a^	1.59	316.0±69.0^a^	1.56	189.4±17.1^c^	54	1428.3±110.1^c^ ^,^ ^d^	34
CPN 2000 μg/plate	23.2±3.7^a^ ^,^ ^b^	0.94	212.7±33.1^a^	1.05	173.5±29.1^c^	59	1497.7±170.7^c^ ^,^ ^d^	31
CPN 5000 μg/plate	10.2±2.5^b^	0.41	112.7±32.3^a^	0.56	166.6±20.9^c^	61	1306.0±174.0^d^	41

^1^ Negative control: 20 μL dimethylsulfoxide (DMSO).

^2^ Positive control: 0.5 μg 4-nitroquinoline 1-oxide (4-NQO)/plate for TA98 and 1.5 μg sodium azide/plate for TA100.

All values are means ± SD of three independent experiments.

^3^ Statistical analysis: one-way ANOVA and the Tukey’s test.

^a, b, c, d^ Letter in common in the same column do not present significant difference (*p* > 0.05). Different letters in the same column present significant difference (*p* < 0.05).

In the evaluation of mutagenicity, the doses of CPN tested (1, 10, 20, 50, 100, 200, 500, and 1000 μg/plate) presented an increase in the number of His^+^ revertants in tester strains TA98 and TA100. Nevertheless, these results did not indicate a significant difference between the negative control and any doses of CPN by one way ANOVA and the Tukey’s test. At the doses of 2000 and 5000 μg/plate, a small decrease in the number of His^+^ revertants was observed for both strains and the results showed a dose-response relation using regression analysis for both strains (*p* < 0.05). However, none of the tested strains reached MI ≥ 2, since this parameter was 1.59 for strain TA98 and 1.56 for strain TA100 at the dose of 1000 μg/plate. There was no significant difference in MI values for TA98 and TA100 strains (*p* > 0.05). Thus, based on the dose-response relation of a large number of doses tested, we can infer that CPN presented a moderate profile of a mutagenic compound. At higher doses, namely 2000 and 5000 μg/plate, CPN showed a toxic effect corroborated by the decrease in the number of His^+^ revertants.

The antimutagenic evaluation demonstrated that all doses of CPN (1, 10, 20, 50, 100, 200, 500, 1000, 2000, and 5000 μg/plate) co-treated with 4-NQO caused a significant decrease in the number of His^+^ revertants in strain TA98 compared to the positive control (4-NQO) (*p* < 0.05). Similarly, a decrease in the number of His^+^ revertants was observed in strain TA100 at all doses of CPN. However, only at doses higher than 50 μg/plate this decrease was significant (*p* < 0.05) compared to the positive control (sodium azide). For both strains, the highest IP was observed at the highest dose of CPN (5000 μg/plate), reaching 60.64% for TA98 and 40.82% for TA100, with a significant difference between them (*p* < 0.05). The results for antimutagenicity suggested that the decrease in the number of His^+^ revertants at higher doses of CPN can be partially attributed to a toxic effect of this compound, already observed in the mutagenic evaluation.

### Mouse bone marrow micronucleus test

Table 3 shows the results of cytotoxic and genotoxic activities in the bone marrow of mice treated with CPN based on MNPCE and PCE/NCE frequency. As expected, the negative control group (DMSO) exhibited low MNPCE frequency, whereas the positive control group showed a significant increase in MNPCE compared to the negative control group (*p* < 0.05), confirming the sensitivity of the micronucleus test.

**Table 3:** Evaluation of cytotoxic and genotoxic activities in the bone marrow of mice treated with sulfonamide chalcone N-{4-[3-(4-nitrophenyl)prop-2-enoyl]phenyl}-benzenesulfonamide (CPN) based on MNPCE and PCE/NCE frequency.

Treatment (mg/kg b.w.)	At 24 h			At 48 h		
	MNPCE/2000 PCE^3^		PCE/NCE^4^	MNPCE/2000 PCE^3^		PCE/NCE^4^
	Mean ± SD	%	Mean ± SD	Mean ± SD	%	Mean ± SD
Negative control^1^	4.2±1.48^a^	0.21	1.04±0.09^a^	4.2±1.48^a^	0.21	1.04±0.09^a^
Positive control^2^	35.4±4.04^d^	1.77	0.64± 0.06^d^	11.0±2.92^c^	0.55	0.47± 0.04^d^
CPN 25	11.2±3.19^b^	0.56	1.05±0.09^a^	7.0±2.35^a^ ^,^ ^c^	0.35	0.97±0.13^a^
CPN 50	18.2±3.27^c^	0.91	0.83±0.08^b^	8.6±1.14^b^ ^,^ ^c^	0.43	0.63±0.04^b^
CPN 100	20.8±2.77^c^	1.04	0.80±0.06^b^	9.2±2.28^b^ ^,^ ^c^	0.46	0.59±0.04^b^ ^,^ ^c^
CPN 150	22.4±3.29^c^	1.12	0.71±0.07^c^	10.2±2.59^b^ ^,^ ^c^	0.51	0.56±0.08^c^

^1^ Negative control: dimethylsulfoxide (DMSO) 0.1 mL/10 g body weight (b.w.).

^2^ Positive control: mitomycin C (MMC) 4 mg/kg (80% LD_50_).

All values are mean ± SD of five mice.

^3^ Statistical analysis: one-way ANOVA and the Tukey’s test.

^4^ Statistical analysis: chi-square (χ^**2**^) test.

Mean values followed by the same letter in the column do not present significant difference at 5% probability.

^a, b, c, d^ Letter in common in the same column do not present significant difference (*p* > 0.05). Different letters in the same column present significant difference (*p* < 0.05).

In the genotoxicity analysis, the groups which received 25, 50, 100, and 150 mg/kg of CPN exhibited mean MNPCE of 11.2, 18.2, 20.8, and 22.4 (per 2000 PCE) at 24 h, and 7.0, 8.6, 9.2, and 10.2 at 48 h. These values correspond to 0.56%, 0.91%, 1.04%, and 1.12% at 24 h and 0.35%, 0.43%, 0.46%, and 0.51% at 48 h, respectively. The negative control group exhibited mean MNPCE of 4.2 (0.21%). At all tested doses, CPN caused an increase in the frequency of MNPCE at 24 h and 48 h compared to the respective negative control group (*p* < 0.05). Only at the dose of 25 mg/kg of CPN, at 48 h, the frequency of MNPCE was not significant (*p* > 0.05). A dose-response effect was observed at both times. These results indicate that CPN showed a genotoxic effect.

The PCE/NCE ratio is a relevant parameter and can be used as an indicator of cytotoxicity. In the groups treated with 25, 50, 100, and 150 mg/kg of CPN, the PCE/NCE ratios were 1.05, 0.83, 0.80, and 0.71 at 24 h, and 0.97, 0.63, 0.59, and 0.56 at 48 h, respectively, whereas for the negative control group this ratio was 1.04 at 24 h and 48 h. These results demonstrate that at 24 h and 48 h the dose of 25 mg/kg of CPN did not cause a significant decrease in the PCE/NCE ratio compared to the negative control group (*p* > 0.05). However, at the doses of 50, 100, and 150 mg/kg at 24 h and 48 h, a significant decrease in the PCE/NCE ratio was observed compared to the negative control group (*p* < 0.05), suggesting a cytotoxic effect of CPN at these doses. The PCE/NCE ratio was lower at 48 h compared to the results obtained at 24 h, indicating that CPN showed higher cytotoxic action at 48 h.

Table 4 shows the results of anticytotoxic and antigenotoxic activities in the bone marrow of mice treated with CPN plus MMC based on MNPCE and PCE/NCE frequency. In the evaluation of antigenotoxicity, the mean MNPCE (per 2000 PCE) results for the groups treated with 25, 50, 100, and 150 mg/kg of CPN + MMC were 20.2, 16.0, 17.8, and 19.6 at 24 h, and 8.0, 7.0, 7.4, and 8.2 at 48 h. These values correspond to 1.01%, 0.80%, 0.89%, and 0.98% at 24 h and 0.40%, 0.35%, 0.37%, and 0.41% at 48 h, respectively. The positive control group presented mean MNPCE of 35.4 (1.77%) at 24 h and 11.0 (0.55%) at 48 h. At all doses, CPN caused a significant decrease in the frequency of MNPCE (*p* < 0.05) at 24 h compared to the respective positive control group. These results indicate that CPN modulated the genotoxic activity of MMC, exhibiting antigenotoxic effect. At 48 h, all doses of CPN induced a smaller attenuation of the genotoxic effect of MMC and the reduction of MNPCE frequency compared to the respective positive group was not significant (*p* > 0.05).

**Table 4:** Evaluation of anticytotoxic and antigenotoxic activities in the bone marrow of mice treated with sulfonamide chalcone N-{4-[3-(4-nitrophenyl)prop-2-enoyl]phenyl}-benzenesulfonamide (CPN) plus mitomycin C (MMC) based on MNPCE and PCE/NCE frequency.

Treatment (mg/kg b.w.)	At 24 h			At 48 h		
	^3^MNPCEs/2000PCEs		^4^PCEs/NCEs	^3^MNPCEs/2000PCEs		^4^PCEs/NCES
	Mean ± SD	%	Mean ± SD	Mean ± SD	%	Mean ± SD
Negative control^1^	4.2±1.48^c^	0.21	1.04±0.09^e^	4.2±1.48^a^	0.21	1.04±0.09^d^
Positive control^2^	35.4±4.04^a^	1.77	0.64± 0.06^a^	11.0±2.92^b^	0.55	0.47±0.04^a^
CPN 25 + MMC	20.2±3.11^b^	1.01	0.67±0.06^a^ ^,^ ^b^	8.0±2.65^a^ ^,^ ^b^	0.40	0.55±0.10^b^
CPN 50 + MMC	16.0±2.55^b^	0.80	0.71±0.08^b^	7.0±1.58^a^ ^,^ ^b^	0.35	0.54±0.06^b^
CPN 100 + MMC	17.8±2.59^b^	0.89	0.80±0.06^c^	7.4±1.52^a^ ^,^ ^b^	0.37	0.58±0.11^b^
CPN 150 + MMC	19.6±3.51^b^	0.98	0.88±0.06^d^	8.2±1.92^a^ ^,^ ^b^	0.41	0.63±0.10^c^

^1^ Negative control: dimethylsulfoxide (DMSO) 0.1 mL/10 g body weight (b.w.)

^2^ Positive control: mitomycin C (MMC) 4 mg/kg (80% LD_50_).

All values are mean ± SD of five mice.

^3^ Statistical analysis: one-way ANOVA and the Tukey’s test.

^4^ Statistical analysis: chi-square (χ^**2**^) test.

Mean values followed by the same letter in the column do not present significant difference at 5% probability.

^a, b, c, d^ Letter in common in the same column do not present significant difference (*p* > 0.05). Different letters in the same column present significant difference (*p* < 0.05).

Regarding CPN anticytotoxic activity, at the doses of 25, 50, 100, and 150 mg/kg of CPN + MMC PCE/NCE ratios were 0.67, 0.71, 0.80, and 0.88 at 24 h and 0.55, 0.54, 0.58, and 0.63 at 48 h, respectively, whereas for the positive control group they were 0.64 at 24 h and 0.47 at 48 h. An increase in PCE/NCE ratio was observed compared to the respective positive control group at all doses of CPN (*p* < 0.05), except for 25 mg/kg at 24 h (*p* > 0.05). The results indicate that the simultaneous treatment attenuated the cytotoxic action of MMC.

## Discussion

Sulfonamide and chalcone are well-known groups which have several pharmaceutical applications in the treatment of a variety of diseases. Recent studies have demonstrated that the combination of these groups also presents important biological activities [19]. Therefore, the present study aimed to evaluate the genotoxicity, cytotoxicity, antigenotoxicity, and anticytotoxicity of CPN using the Ames mutagenicity test and the mouse bone marrow micronucleus test. These tests are the most frequently used and recommended by regulatory agencies for determining the genetic risk of various types of substances [11].

Based on the results using the Ames test, CPN presented a moderate profile of a mutagenic and toxic compound. The toxic effect of this compound could be related to an antibacterial effect of sulfonamides, which are structurally similar to p-aminobenzoic acid (PABA), a precursor in folic acid biosynthesis, which in turn is a precursor of nucleic acids. Thus, sulfonamides prevent the incorporation of PABA into the folic acid molecule by competing with it in the active site of dihydropteroate synthase. As a consequence, non-functional analogs of folic acid are formed, inhibiting bacterial cell growth [20,21]. This possible bactericidal property of CPN may result in false negative results and/or underestimation of its mutagenicity using the Ames test. This can be justified by the low values of MI observed after the treatment with higher doses of CPN.

In the micronucleus test, the treatment with CPN induced a significant increase in MNPCE at 24 h and 48 h (*p* < 0.05), demonstrating its genotoxic action. A significant decrease in PCE/NCE ratio was observed at higher doses of CPN at 24 h and 48 h (*p* < 0.05) compared to the negative control, which suggests that CPN presented cytotoxic effect.

The genotoxic and cytotoxic activities of CPN might possibly be attributed to the presence of a nitro group. It is known that nitro chalcones present higher mutagenic and carcinogenic potential compared to non-substituted chalcones [22]. Substituents on aromatic B-rings at C(4) position, such as the nitro group in CPN, are essential for these activities [23]. Compounds that have a nitro group present genotoxic and cytotoxic activities possibly associated with enzymatic reduction, which in turn generates nitrous intermediates and N-hydroxylated metabolites [22]. N-hydroxylated intermediates react with thiol groups, which are present in proteins, and by acetylation become promoters of reactions related to DNA adduct formation, resulting in chromosomal instability [24].

Studies revealed that chalcones and sulfonamides can also inhibit tubulin formation [25,26]. This could explain the anti-mitotic activity of chalcone derivatives, by inhibiting tubulin polymerization and preventing the formation of mitotic spindle [25]. Structure-activity relationship (SAR) studies showed that aryl sulfonamide derivatives inhibit tubulin polymerization via hydrophobic interaction. Moreover, this interaction occurs between the oxygen atom of the sulfonamide portion and the amino hydrogen present in tubulin lysine residues [26,27]. However, when the inhibition is associated with other types of cell damage, caused by mutagenic processes, cells can evade apoptosis by forming aneuploid or micronuclei cells [28–30]. Therefore, the genotoxic and cytotoxic actions of CPN can also be related to the inhibition of tubulin formation.

According to the results of the antimutagenic evaluation, CPN displayed considerable antimutagenic activity in both assays applied in this study, Ames and micronucleus tests. Furthermore, using the micronucleus test, CPN showed an increase in PCE/NCE ratio compared to the respective positive control, indicating a protective effect against the cytotoxic action of MMC in mice bone marrow.

The antimutagenic action of CPN observed in the Ames test could be partially attributed to its antibacterial effect at higher doses, corroborated by the results of the mutagenic evaluation using the same test in the present study. Previous studies have shown that chalcone derivatives presenting α,β-unsaturated ketones exhibited antimutagenic effect against the promutagen benzo(a)-pyrene [4]. Thus, the chemoprotective effect exhibited by CPN in both assays could be attributed, at least partially, to the presence of an α,β-unsaturated portion in this compound.

Our results indicate that CPN presented genotoxic and antigenotoxic actions. Many antimutagenic substances have also been proven to be mutagenic. For instance, quercetin is converted into a potentially toxic product while offering protection by scavenging of reactive oxygen species (ROS) in the cell [31]. Additionally, a number of substances which present antimutagenic and/or anticarcinogenic actions against heterocyclic amines also exhibit mutagenic or carcinogenic responses [32]. Most of these modulating substances come from plants and so do the chalcones.

In conclusion, CPN presented genotoxic, cytotoxic, antimutagenic, antigenotoxic, and anticytotoxic activities under the experimental conditions applied in this study. However, more studies are required to better understand the mechanisms of DNA damage as well as the protective action of CPN to evaluate its possible use as a therapeutic agent.
